# Autophagy in endothelial cells and tumor angiogenesis

**DOI:** 10.1038/s41418-019-0287-8

**Published:** 2019-01-28

**Authors:** Marco B. Schaaf, Diede Houbaert, Odeta Meçe, Patrizia Agostinis

**Affiliations:** Cell Death Research & Therapy (CDRT) Laboratory, Department for Cellular and Molecular Medicine, KU Leuven University of Leuven, Leuven, Belgium

**Keywords:** Cancer microenvironment, Tumour angiogenesis, Macroautophagy

## Abstract

In mammalian cells, autophagy is the major pathway for the degradation and recycling of obsolete and potentially noxious cytoplasmic materials, including proteins, lipids, and whole organelles, through the lysosomes. Autophagy maintains cellular and tissue homeostasis and provides a mechanism to adapt to extracellular cues and metabolic stressors. Emerging evidence unravels a critical function of autophagy in endothelial cells (ECs), the major components of the blood vasculature, which delivers nutrients and oxygen to the parenchymal tissue. EC-intrinsic autophagy modulates the response of ECs to various metabolic stressors and has a fundamental role in redox homeostasis and EC plasticity. In recent years moreover, genetic evidence suggests that autophagy regulates pathological angiogenesis, a hallmark of solid tumors. In the hypoxic, nutrient-deprived, and pro-angiogenic tumor microenvironment, heightened autophagy in the blood vessels is emerging as a critical mechanism enabling ECs to dynamically accommodate their higher bioenergetics demands to the extracellular environment and connect with other components of the tumor stroma through paracrine signaling. In this review, we provide an overview of the major cellular mechanisms regulated by autophagy in ECs and discuss their potential role in tumor angiogenesis, tumor growth, and response to anticancer therapy.

## Abstract (web summary)

Vascular homeostasis relies on the proper behavior of endothelial cells (ECs). Emerging evidence indicate a critical role of autophagy, a vesicular process for lysosomal degradation of cytoplasmic content, in EC biology. While EC-intrinsic autophagy promotes EC function and quiescent state through redox homeostasis and possibly metabolic control, a role for EC-associated autophagy in cancer seems more complex.

## Graphical abstract

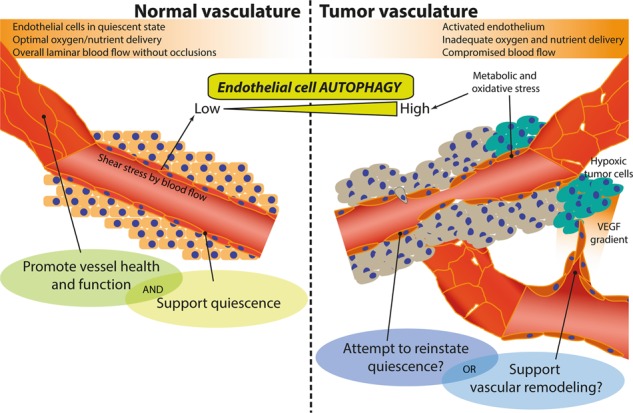

## Facts

- EC-intrinsic autophagy is inherently cytoprotective and regulates its function in response to blood flow and metabolic stress.
- EC-intrinsic autophagy is crucial for redox homeostasis and vessel permeability.
- Autophagy in EC is compromised during aging.
- Autophagy in EC interfaces with metabolic pathways and lipid homeostasis.
- Tumor hypoxia and metabolic stress stimulate autophagy in the tumor vasculature.
- In solid tumors EC-specific deletion of autophagy genes fosters unproductive angiogenesis.
- The autophagy blocker chloroquine induces vessel normalization in tumors independent on key autophagy mediators.

## Open questions

- Does EC-intrinsic autophagy influence EC plasticity and specification?
- Does compromised EC-intrinsic autophagy contribute to features of the aged vasculature?
- Which are the EC secreted factors depending on intrinsic autophagy?
- How does specific blockade of EC-intrinsic autophagy impact tumor angiogenesis?
- Is there a different role for autophagy in normal ECs versus tumoral ECs?
- How does tumor vessel-associated autophagy shape the tumor microenvironment?
- Can the EC-associated autophagy/endo-lysosomal pathways offer novel therapeutic targets to improve vessel functions and anticancer therapies?

## Autophagy: a brief introduction

Autophagy, or self-eating, is an evolutionary conserved mechanism enabling the delivery and/or direct targeting of cytoplasmic materials to the lysosome for degradation and recycling. In recent years autophagy has emerged as one of the major cellular adaptation pathways with expanding cell-autonomous and non-autonomous functions, including but not limited to, redox control, metabolism, and unconventional secretion, thus controlling tissue/organism—rather than only cellular—homeostasis. Autophagy has key roles in development and differentiation, and, not surprisingly, autophagy defects underlie various disorders including neurodegeneration, metabolic diseases, infectious diseases, and cancer [1].

**Fig. 1:**
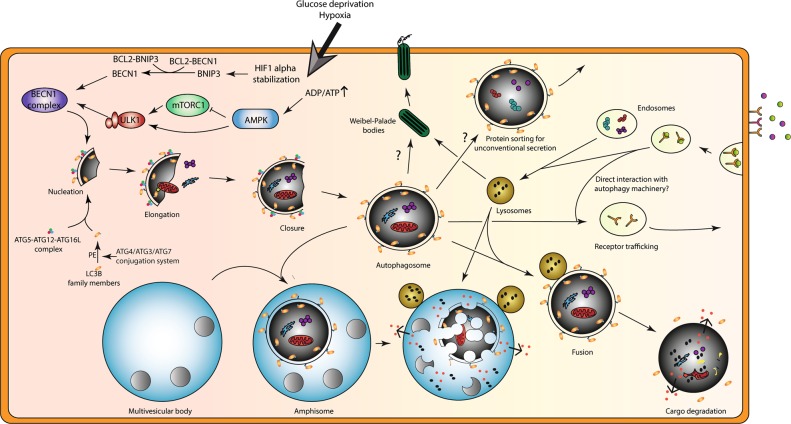
Autophagy induction during metabolic stress and its proposed functions beyond (targeted) lysosomal degradation in endothelial cells. Autophagy and its related proteins are implicated in additional cellular processes that control endothelial cell (EC) behavior. At least in part this may be due to the close interplay of autophagy machinery with components of the endo-lysosomal system. Yet, it must be noted that both autophagy and endosomes/lysosomes also control distinct processes as will be clarified throughout this review. Top left: basal level activity of autophagy is stimulated upon metabolic stresses that induce signaling events to increase the formation of the initial cup-shaped membrane called a phagophore (nucleation). At the phagophore membrane, a complex consisting of class III PI3K, VPS34, and Beclin1 (BECN1) generates PI3P, thereby facilitating recruitment of the ATG12-ATG5-ATG16L complex. The latter facilitates the conjugation of LC3B (or other family members) to phosphatidylethanolamine (PE) for membrane anchoring of this key autophagy marker. Metabolic stressors (e.g. hypoxia, causing stabilization of hypoxia-inducible factor (HIF), glucose deprivation, leading to activation of AMPK and inhibition of mTOR) stimulate autophagy in ECs. HIF induces expression of BNIP3 that competes with BECN1 for BCL2 interaction thereby releasing BECN1. Reduced activity of mTORC1 relieves ULK1 complex from inhibition that can subsequently induce autophagy. (Diagonal) The phagophore further elongates to form a closed autophagosome, which engulfs cytoplasmic constituents (non)specifically. After fusion with a lysosome, the autophagosomal content is degraded releasing metabolites that are recycled by the cell. Bottom left: a connection between autophagosomes and multivesicular bodies (collection of luminal vesicles) exists in which fusion events generate a so called amphisome. Top middle: autophagy as well as lysosomal machinery are required for proper formation of Weibel–Palade bodies (storage/secretory granules of ECs). Top right: in addition, key autophagy proteins and autophagosomes are implicated in unconventional secretion of proteins, which bypasses the classical endoplasmic reticulum-to-Golgi route. Here autophagy may serve as a vesicular mechanism for protein transport across the plasma membrane or receptor trafficking for cell surface localization. Right: endosomal vesicles regulate receptor cell surface localization as well as protein secretion events

Autophagy is known to exist in three different primarily mechanisms; chaperone-mediated autophagy, which entails the recognition of a KFERQ-like motif in damaged/aberrant proteins by cytosolic heat shock cognate 70 chaperone and their translocation to the lysosome through the lysosomal LAMP2A receptor (reviewed in [2]); microautophagy, in which cargo is directly internalized by the lysosomes/late endosomes (reviewed in [3]), and macroautophagy, in which the cytoplasmic cargo is captured into a double-membrane vesicle (autophagosome) that is trafficked to and ultimately fuses with lysosome for degradation (depicted schematically in Fig. 1). In all cases, the end products of these degradation systems are recycled into the cytosol and are reused in various processes including protein, lipid, and nucleotide synthesis and ATP production.

Among these different variants, macroautophagy (hereafter referred to as autophagy) is the best studied lysosomal pathway for recycling of intracellular components, including potentially toxic or damaged/superfluous macromolecules, such as proteins and lipids, as well as whole organelles. Autophagy is thus unique among other lysosomal pathways of degradation because it is the only mechanism that involves the formation of autophagosomes, for cargo delivery to the lysosomes. Under physiological conditions, at basal, low-level autophagy functions as major cytoplasmic quality control and stress-adaptation mechanism, thus ensuring cellular homeostasis. In response to various stimuli, including stress and extracellular cues, autophagy is activated in an attempt to promote survival during adverse conditions. As such, metabolic stresses (nutrient deprivation and hypoxia) or oxidative stress induce autophagy in order to increase macromolecule recycling (to fuel metabolism or protein production) or degrade potentially toxic reactive oxygen species (ROS)-producing organelle (commonly mitochondria). Autophagy is therefore inherently cytoprotective and several studies have indeed confirmed that pharmacological or genetic inhibition of autophagy curtails its pro-survival ability and induces cell demise.

At the molecular level autophagy involves the coordinated action of a conserved set of autophagy-related genes (*Atg*), which control various stages of the process in a hierarchical manner, that is, the initiation of autophagosome formation, its elongation, trafficking, and fusion with the lysosomes (see Fig. 1 for more details, and for a complete view of molecular autophagy readers are referred to [4]). Initially, autophagy was believed to be an unselective bulk degradation process typically stimulated under conditions of nutrient deprivation, to support the cell’s bioenergetics needs and survival. However, we now know that this catabolic process can be highly specific depending on the type of initiation stimulus or insult. Clearance of mitochondria, through mitophagy, or degradation of protein aggregates that cannot be removed by the proteasome, through aggrephagy, are examples of selective autophagy pathways with increasing implications in physiological and pathological conditions [5].

Moreover, besides their crucial implication in canonical autophagy, a subset of Atg players or circuits are emerging as mediators of various trafficking processes (for more detail authors refer to [6]) (Fig. 1). This is perhaps not surprising considering that multiple yeast orthologs and splice variants of certain autophagy genes exist (i.e. LC3B, LC3A (two splice variants), LC3C, GABARAP, GABARAPL1, and GABARAPL2) that are implicated in specialized roles of the autophagy pathway (e.g. selective targets for autophagy, involvement in specific steps in autophagosome/autolysosome maturation) or receptor trafficking [7]. In addition, accumulating evidence implicates various *Atg* genes in broader vesicular trafficking processes, such as endocytosis [8, 9], phagocytosis [10, 11], exocytosis [12], and unconventional secretion (that is independent on the classical endoplasmic reticulum-to-Golgi anterograde transport system) [13, 14], which is emerging as a key process regulating intercellular cross talk especially in the context of cancer, as discussed below. Altogether, these examples demonstrate that the homeostatic role of autophagy and its related proteins is more elaborate than originally thought and goes well beyond the degradation of cytoplasmic content alone.

This complexity is also reflected in endothelial cells (ECs), the main cellular constituents of the vascular system in vertebrates.

## Autophagy in ECs

In higher vertebrates ECs construct the inner lining of all subvascular compartments, which supply nutrients and oxygen to all distal tissues therefore maintaining tissue/organism health and homeostasis (readers are referred to excellent reviews [15–17] for a more detailed overview on vascular development and specifications). Vascular homeostasis relies heavily on proper behavior of ECs (described in further sections and Fig. 3) and therefore, not surprisingly alterations of main EC’s biological function caused by pathological insults or aging processes are linked to a variety of diseases including, but not limited to, atherosclerosis [18, 19], neurodegenerative disorders [20], and cancer [21].

The role of autophagy in ECs has been explored in more detail only in recent years. An emerging body of literature implicates vascular autophagy in prenatal vascular development and several age-related vascular pathologies. In line, autophagy supports vascular development during embryogenesis [22] and expression of key autophagy proteins (ATG7, ATG8, and Beclin1 (BECN1)) in the angiogenic plexus vessels associates with EC–EC junctions to prevent hemorrhaging [23]. In healthy organisms, ECs are mostly found in a quiescent state but retain the ability to dynamically respond to microenvironmental changes or stimuli. These include pro-angiogenic cues to form new blood vessels from pre-existing vessels (angiogenesis) [24] or low oxygen and nutrient availability. Thereafter, ECs return quiescent upon vessel perfusion and restoration of physiological levels of oxygen and nutrients.

Recent studies suggest that autophagy may serve as a dynamic mechanism enabling ECs to adjust their bioenergetic and biosynthetic needs in response to the changing environment, presence of angiogenic cues, or intrinsic and extrinsic insults or injuries (as discussed later). In contrast, deregulated autophagy upon, e.g., prolonged induction of nutrient deprivation or depletion of growth signals, may be detrimental to EC function and can lead to autophagic cell death [25–27]. However, the exact role that autophagy plays in modulating EC responses is still controversial and likely dependent on the type of metabolic stress or the experimental conditions used in various studies.

Concomitant to the age-related increased risk of cardiovascular disease, the autophagy machinery is compromised in the aged endothelial compartment [28]. In line with this, in older mice ECs display lower levels of key pro-autophagic proteins, such as BECN1 and LC3, compared to younger animals [29]. Fundamental to age-related cardiovascular diseases are increased oxidative stress and impaired endothelium-dependent dilatation, due to reduced availability of the key vasodilator mediator nitric oxide (NO) produced by ECs [30]. As it will be discussed in the next sections, autophagy is a crucial regulator of both processes, thereby linking this catabolic pathway to maintenance of key homeostatic endothelial function. The protective benefit of autophagy in preserving vascular homeostasis is relevant as activation of autophagy by caloric restriction can modulate longevity by slowing down vascular aging [31]. Nevertheless, autophagy in ECs is commonly stimulated under pathological conditions, where an increased autophagic flux may merely be an attempt to repair the insult or reinstate EC homeostasis to either counteract disease progression. In particular, emerging in vivo studies delineate an important role of EC-associated autophagy in cancer, a life-threatening disease hallmarked by a continuous remodeling of the vasculature, which represents a main route for cancer cell dissemination, modulation of antitumor immunity, and therapy responses.

In this essay, we will first briefly discuss current knowledge on the physiological role of autophagy in EC biology. Thereafter, we will discuss recent studies unveiling a role for autophagy/lysosomal pathways of tumor-residing ECs in angiogenesis, cancer cell dissemination, and therapeutic responses.

### EC autophagy as key regulator of redox homeostasis and quiescence

Mechanisms keeping EC quiescence in check are important for EC survival and to maintain vessels in a matured state. A critical modulator of EC biology and EC fate is the redox system. A low degree of oxidative stress is thought to promote proper EC function and maintain the quiescent phenotype of ECs. In contrast, deregulated ROS levels in ECs promote angiogenesis and may become toxic [32].

**Fig. 2:**
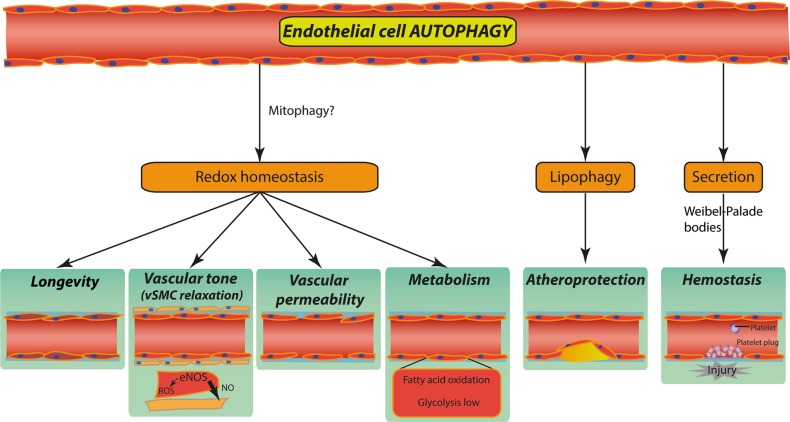
Proposed roles of autophagy in endothelial cells (ECs). Proficient EC autophagy is associated with low-level oxidative stress and thus contributes to redox homeostasis thereby regulating and interfacing with key reactive oxygen species (ROS)-mediated EC function/behavior including: (1) vascular aging thus supporting longevity; (2) nitric oxide (NO) production and vasodilatation; (3) endothelial barrier integrity and vascular permeability; (4) the SIRT1/FOXO1/MYC signaling, with implications for fatty acid (FA) uptake and oxidation through mitophagy; (5) lipid homeostasis through lipid droplet degradation (lipophagy), which protects against atherosclerotic plaque formation; (6) hemostasis by regulating the secretion of EC von Willebrand factor from Weibel–Palade bodies (see the main text for more details)

The maintenance of a functional EC redox state is emerging as a key cell-autonomous mechanism regulated by autophagy (Fig. 2). However, the link between EC redox state and autophagy is complex as both pathways are intimately linked and interconnected with other crucial processes, like metabolism, that orchestrate EC plasticity and angiogenesis. Under physiological conditions, laminar shear stress, a known inducer of ROS production, stimulates autophagy in ECs, which is relevant for their survival [33, 34]. Interestingly, stimulation of autophagy by laminar flow appears to be dependent on the flow-induced activity of the NAD^+^-dependent histone deacetylase Sirtuin 1 (SIRT1) [34]. Under flow-induced oxidative stress, SIRT1 is upregulated and stimulates autophagy by directly deacetylating crucial pro-autophagic proteins [33, 34] and by promoting the transcription of several components of the autophagy machinery such as *ATG5*, *BECN1*, and *LC3* [34]. This transcriptional regulation is thought to involve SIRT1-mediated deacetylation of the Forkhead box O1 transcription factor (FOXO1), resulting in the selective upregulation of a subset of autophagy genes in ECs in response to shear stress. Furthermore, in ECs exposed to flow, shear stress-induced SIRT1/FOXO1 signaling suppresses MYC to maintain quiescence and limit EC proliferation [35] (Fig. 2). Notably, FOXO1 under homeostatic conditions restricts EC migration and angiogenesis [35] and is a positive modulator of autophagy, through mechanisms that involve, besides its nuclear activity, also its cytoplasmic localization and direct binding to ATG7 [36]. Interestingly, depletion of cell-autonomous vascular endothelial growth factor (VEGF) in ECs increases FOXO1 activation both in vitro and in vivo, leading to mitochondrial fragmentation and bioenergetics defects causing ultimately autophagic cell death [25]. These findings suggest that the upstream stressors and mechanisms mediating FOXO1-dependent activation of autophagy may ultimately regulate EC fate.

How autophagy exactly contributes to the maintenance of a quiescent EC phenotype is not completely understood. Nonetheless, preserving cytoplasmic quality control and regulating bioenergetics, through the removal of damaged and ROS-producing mitochondria, could contribute to the intrinsic beneficial effect of EC-associated autophagy (Fig. 2). Mitophagy, involving PTEN-induced kinase 1 and PARKIN, is activated in response to metabolic stress in ECs and prevents mitochondrial dysfunction and metabolic stress-induced endothelial injury [37]. Hence stress-induced EC autophagy/mitophagy in ECs could exert angiostatic effects, while their inhibition might shift ROS production and signaling to pro-angiogenic or lethal levels. This theory would be consistent with the in vivo anti-angiogenic effects of the C-terminal domain of heparan sulfate proteoglycan 2 [38] and the mTOR inhibitor torin, which stimulate autophagy in ECs [39]. Additionally, the angiostatic effects of the secreted proteoglycan decorin, which depends on PEG3-dependent transactivation of thrombospondin 1, is thought to rely on autophagy activation in ECs [40]. However, these studies only indirectly link EC-intrinsic autophagy to the angiostatic effects of decorin or mTOR inhibition, and should be validated by genetic disruption of autophagy genes in these settings.

The EC redox state is also a key determinant of vascular permeability [41] and EC-intrinsic autophagy maintains low permeability by limiting ROS production, thereby sustaining barrier integrity [42] (Fig. 2). In ECs, genetic or pharmacological inhibition of autophagy/lysosomal function through small interfering RNA targeting of ATG5 or BafilomycinA1, respectively, increases ROS formation and EC permeability, which can be partially rescued by antioxidants. Systematic, in-depth proteomics studies further show that ECs require autophagy to maintain endothelial barrier integrity [42]. *Atg3*-deficient ECs have impaired endothelial NO synthase (eNOS) phosphorylation and are incapable of inducing NO in response to shear stress [43]. NO has an essential cellular signaling role in vasodilatation and angiogenesis, with low concentrations of NO being pro-angiogenic whilst high concentrations are inhibitory. This further underscores a potential role for key autophagy factors in modulating these crucial endothelial processes. Of note, in the aged endothelium, ROS is increased and ROS scavenging mechanisms are impaired [44]. This endothelial dysfunction is further aggravated by a ROS-mediated change in the activity of eNOS, from a NO-producing to a superoxide anion-producing enzyme, which results in impaired NO generation [45]. Although not proven, it is tempting to speculate that the decreased autophagic flux observed in the aged endothelium may contribute to the deleterious effects of ROS accumulation in ECs.

In conclusion, basal “low-level” autophagy under physiological stress conditions may support EC’s quiescent state and sustain barrier integrity, through its essential role in the regulation of redox homeostasis. However, a clear mechanistic view of the signaling processes regulated by autophagy in ECs is still missing.

### EC autophagy in metabolism and lipid homeostasis

Normal EC function protects vessels against the formation of atherosclerotic lesions. Mounting evidence shows that autophagy has a vital role in atherosclerogenesis. In a murine atherosclerotic model, genetic inactivation of EC autophagy increases plaque burden exclusively in high shear stress areas that are normally resistant to atherosclerotic plaque formation [27, 46]. This indicates that autophagy is essential for atheroprotection by restoring endothelial vascular wear and tear under physiological blood flow. Interestingly, intracellular lipid accumulation in incipient atherosclerotic lesions, suggests that lipophagy—a specialized autophagy pathway targeting lipid droplets [47] may be crucial to maintain vascular function (Fig. 2). In lipophagy, intracellular lipid depots or droplets consisting of triglycerides are targeted for acid hydrolase digestion and selective degradation in the lysosomal compartment, thereby yielding free fatty acids (FAs). It is noteworthy that low-density lipoprotein (LDL) exposure induces autophagy in ECs, and EC-specific *Atg7*-deficient mice retain higher LDL levels with an increased atherosclerotic burden compared to wild-type (WT) control mice [48]. Together, these studies suggest that autophagy-mediated lipid homeostasis promotes vascular function, a connection that needs further experimental validation.

**Fig. 3:**
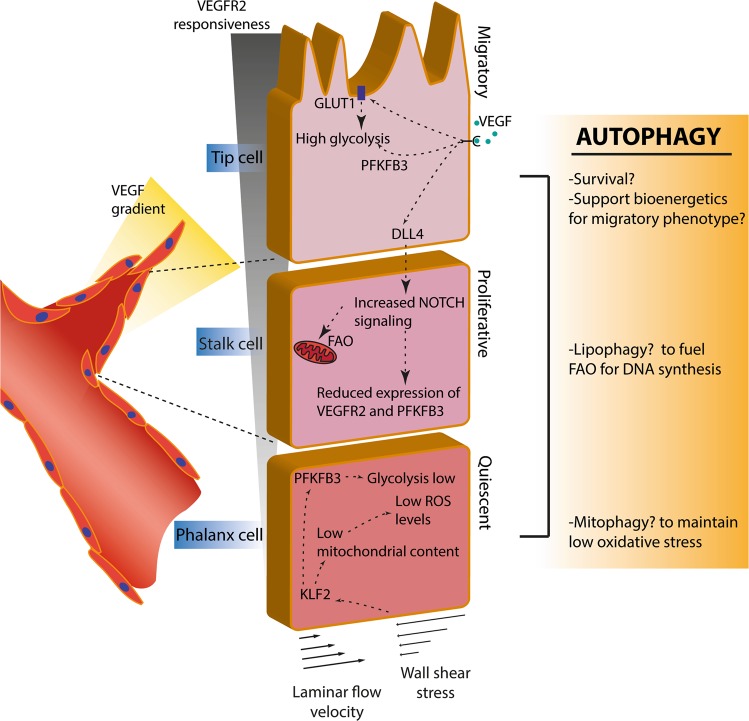
Regulation of distinct endothelial cell (EC) phenotypes during vessel sprouting. The vascular endothelium consists of three main EC subtypes with specialized morphological and functional features. In adults, ECs are mostly found in a non-proliferating, quiescent state (phalanx cells), yet are readily able to respond to external cues and initiate angiogenesis (the formation of new blood vessels from pre-existing vessels). This process entails the differentiation of ECs to guide the growing sprout or branch. Pro-angiogenic signals as vascular endothelial growth factor (VEGF) isoforms that bind to their receptor (VEGFR) stimulate EC migration, proliferation and sprouting. In ECs, VEGF binding to VEGFR2 signals to induce a sprouting migratory phenotype or a proliferating phenotype, referred to as tip or stalk cells, respectively. In brief, the activation of the VEGF/VEGFR2 axis in the tip cell induces the expression of the NOTCH ligand the Delta-like ligand (DLL)4 along with the upregulation of the rate-limiting glycolytic enzyme 6-phosphofructo-2-kinase/fructose-2,6-biphosphatase 3 (PFKFB3) and glucose uptake, via the glucose transporter GLUT1. In the tip cell increased glycolysis fuels the cytoskeleton remodeling at lamellipodia and lobopodia, thereby supporting the migratory phenotype. DLL4 interaction with the NOTCH receptor on neighboring ECs leads to the proteolytic activation of the transcription factor Notch intracellular domain (NICD). NICD represses PFKFB3 and VEGFR2 expression while increasing fatty acid oxidation (FAO) that is required for DNA replication, thus supporting the proliferative stalk phenotype. In contrast, phalanx cells are kept quiescent due to laminar shear stress-induced Krüppel-like factor 2 (KLF2). KLF2 in turn, represses PFKFB3 expression, reduces proliferation, and causes reduction of mitochondrial content. Autophagy may be involved in the regulation of these subtypes through e.g. increasing the resistance to cell death upon e.g. hypoxic conditions, and by sustaining the high energy demand of tip and stalk cells through the modulation of metabolic pathways in these ECs. Stimulation of autophagy/mitophagy by laminar flow in phalanx cells maintains redox homeostasis to preserve EC quiescence

Moreover, recent studies would support a molecular link between autophagy and EC metabolism, which is emerging as a major molecular and cell-autonomous regulatory trait of EC specification and angiogenesis (Fig. 3).

Although ECs have immediate access to oxygen in blood and maintain the ability of mitochondria respiration, ECs dynamically use glycolysis as major metabolic route for ATP production and redox control [49]. However, rewinding of metabolic pathways in ECs is linked to their specification. High glycolytic activity is a major characteristic of tip cells, which are exposed to higher VEGF concentration. Indeed, EC glycolysis is highly increased in response to pro-angiogenic stimuli, fostering endothelial migration. The VEGF-VEFGR2 axis promotes glycolysis in proliferating ECs by upregulating the rate-limiting glycolytic enzyme 6-phosphofructo-2-kinase/fructose-2,6-biphosphatase 3 (PFKFB3) and glucose uptake via the glucose transporter GLUT1 (Fig. 3). This enables cytoskeleton F-actin remodeling during EC migration by locally increasing ATP availability at the lamellipodia and lobopodia. In the highly proliferative stalk cells, anti-angiogenic NOTCH signaling decreases glycolytic flux by downregulating PFKFB3 levels [49]. In these hyper-proliferating ECs, FA oxidation (FAO) represents a major fuel driving proliferation and DNA synthesis. Recent elegant in vivo studies underscore that FAO finely modulates stalk cell behavior [50], and compromising FAO in these ECs also induces EC hyper-permeability [51]. While the role of autophagy in modulating this metabolic route is yet to be explored, lipophagic output of free FAs may fuel mitochondrial β-oxidation in these highly proliferating cells, along with other anaplerotic substrates, such as glutamine, whose availability is known to be regulated by autophagy [52–54]. How metabolism is rewired in quiescent phalanx cells is not completely understood. Global metabolic profiling in quiescent ECs underscores an upregulation of FAO gene sets, and a lower expression of glycolysis and oxidative phosphorylation genes [50]. An interesting possibility entails that FAO promotes EC quiescence by controlling redox homeostasis through enhanced NADPH production [50]. Of note, the anti-proliferative effects of laminar shear stress-induced Krüppel-like factor 2 (KLF2) or its overexpression in ECs, is accompanied by reduced mitochondrial mass and decreased glycolytic flux [55] (Fig. 3).

Whether autophagy may contribute to promote EC quiescence through lipid droplet degradation and increased FAO is not known. Interestingly, in aging mice, a reduction in SIRT1 activity in ECs is associated to reduced FA uptake and oxidation, mitochondrial impairment, and increased oxidative stress [56], causing an overall impairment of EC function. Given the role of SIRT1 and redox signaling in the transcriptional control of autophagy genes in ECs [34], these findings further underpin a possible link between autophagy and FAO metabolism, a hypothesis warranting further investigations. While this interesting connection still needs to be validated, under conditions of excessive accumulation of FAs (i.e. palmitic acid) that cause EC dysfunction, autophagy may become toxic and promote lipoxic signaling leading to necroptosis [57].

In summary, these studies suggest that autophagy in ECs may contribute to the metabolic control of EC fate specification, an interesting connection urging future validation.

### EC autophagy/lysosomal pathways and regulation of receptor signaling and secretion

Intercellular communication is achieved through ligand-receptor interactions involving surface-bound or secreted proteins that act on target cells in an auto- or paracrine manner. The spatio-temporal duration and intensity of receptor-mediated signaling events are regulated through the endocytic route and the lysosomes [58]. In ECs these are illustrated by endocytosis of VEGFR2 that protects the receptor from cleavage [59] and turnover of the NOTCH receptor to control plasticity in tip/stalk cell phenotype. VEGFR2 internalization and turnover are reduced in the more quiescent and mature vessel plexus [60] supporting a mechanism of preservation of the resting state. Moreover, quiescent ECs establish bidirectional connections through junctional molecules like vascular endothelial-cadherin (CDH5), which strengthen their barrier function, and intercellularly, with pericytes, which are required to promote vessel stabilization. Notably, these cell-to-cell interactions implicate an important role for vesicular trafficking mechanisms in controlling EC behavior [61]. Altering the acidic pH of the late endosome or lysosomes by chloroquine (CQ), which compromises fusion events and disrupts endosomal and autophagic cargo degradation, results in the activation of NOTCH1 signaling through the endocytic route, and consequent NOTCH1 intracellular domain-mediated EC quiescence [62]. Interestingly, this CQ effect is not phenocopied by loss of ATG5. Whether these differential effects extend to other crucial EC surface receptors, such as the VEGFR1 and VEGFR2, and affect their downstream signaling is currently not known.

Beyond the regulation of receptor availability via the endocytic route, autophagy genes in EC appear to have a fundamental role in secretion. Endothelial secretory granules called Weibel–Palade bodies (WPBs) contain active molecules, including von Willebrand factor (VWF), P-selectin, interleukin-8, angiopoietin2, and endothelin1 (Fig. 2). ECs contribute to the regulation of coagulation and fibrinolysis by expressing a variety of molecules regulating the activation of platelets and the coagulation cascade, thus preventing thrombus formation after vessel injury [63]. Endothelial-specific conditional deletion of *Atg7* or *Atg5* in mice does not affect vessel structure or capillary density, but limits VWF release upon epinephrine stimulation and consequently causes prolonged bleeding time. These EC-intrinsic autophagy-mediated effects are caused by the incorrect processing and secretion of VWF via the WPBs in response to epinephrine [64]. Of note, in vitro, silencing of BECN1 does not affect VWF exocytosis, whereas ATG5 or ATG7 knockdown or CQ treatment do, suggesting a specific contribution of these specific autophagy mediators and the endo-lysosomal system in VWF secretion. In this context, it is remarkable that KLF2 expression changes the composition of WPBs, by reducing angiopoietin2 content of WPBs [65]. Secreted angiopoietin2 is a main destabilizer of EC-perivascular cell interactions, as it antagonizes angiopoietin1/TIE2-induced vessel stabilization [66].

Thus, shear stress-induced KLF2 and autophagy may congregate to regulate EC secretory profile thereby preserving the maturation state and function of vessels, an interesting connection that needs to be experimentally validated in future studies.

## Autophagy in the tumor endothelium

In cancer, autophagy is considered a double-edged sword [67]; i.e. in a pre-malignant stage, autophagy plays an oncosuppressive role by maintaining cytoplasmic quality control and homeostasis through degradation of cytotoxic constituents or oncogenes, by activating cellular senescence or by limiting inflammation, among other potential tumorigenic events (reviewed in [68]). Once a tumor is formed, autophagy contributes to survival of cancer cells in areas deprived of nutrients or oxygen (hypoxia) [69], a common feature of solid tumors that contributes to tumor progression, therapy resistance, and metastasis formation [70]. In established tumors, elevated levels of autophagy are often found associated to poorly oxygenated regions where the demand for nutrients and the need to withstand several forms of metabolic stress in order to survive, are increased [68, 69]. Several advanced tumors display an “autophagy addiction”, which appears to be required to maintain their energy balance, through the recycling of intracellular components into biosynthetic pathways or ATP synthesis and to regulate secretion of pro-tumorigenic factors [13, 71]. In line with this notion, in the context of advanced and aggressive tumors such as pancreatic cancer, autophagy is hijacked by oncogenes to support energy metabolism and allow growth under conditions of energy deficit and metabolic stress [72, 73].

Another emerging aspect linking autophagy to tumor progression is the ability of malignant cells to use autophagy as a trafficking and export mechanism of pro-tumorigenic factors, such as pro-inflammatory/pro-angiogenic cytokines or chemotactic/pro-invasive molecules, such as extracellular ATP [13, 74, 75]. However, apart from cancer cells, the tumor microenvironment (TME) of a solid tumor contains a complex interstitial extracellular matrix and various stromal cells. These cells are recruited from the surrounding tissues or from the bone marrow and include fibroblasts, cells of the immune systems, pericytes, and ECs of the blood and lymphatic vasculature. It is now increasingly accepted that the interface between malignant and non-transformed cells within the TME represents a highly plastic tumor ecosystem that supports tumor growth and dissemination through the various stages of carcinogenesis.

In spite of this, whereas the role of autophagy in cancer cells is well-studied, its role in the tumor stroma is far from being understood. Some recent elegant studies have provided evidence for the differential role of autophagy mediators in cancer cell or stromal cells in regulating the TME and tumor control (reviewed in [67, 76]). For example, genetic loss of cancer cell autophagy and subsequent p62 accumulation leads to a chronic pro-inflammatory and pro-angiogenic microenvironment that assists tumor initiation and progression. Oppositely, increased expression of p62 in cancer-associated fibroblasts through blockade of autophagy reduces IL-6 secretion and is overall anti-inflammatory [77, 78]. This example indicates the necessity to clarify the role of autophagy in shaping the cross talk between cancer cells and stromal cells in order to gain key insights in how autophagy intervention could impact stroma cell function, for better or worse regarding tumor development and therapy outcome. In particular, studying the role of autophagy in the vascular compartment seems of vital relevance since the (aberrant) tumor vasculature provides not only a way to replenish nutrients to starved cancer cells, but represents a major escape route for the stressed cancer cells. Moreover, the tumor vasculature is crucially involved in the trafficking and activity of immune cells, thereby contributing to immunosurveillance mechanisms.

In the next sections we will discuss some of the emerging features highlighting a role for autophagy and the endo-lysosomal system in tumor ECs (TECs) and cancer progression. We moreover discuss whether harnessing EC autophagy/lysosomal pathway in tumor might be of therapeutic value in anticancer therapy.

### Tumor-associated vasculature is in a state of continuous remodeling

In solid tumors, angiogenesis is induced to receive nutrients (e.g. oxygen and glucose) required for cancer cell’s high energy demand and growth. Tumor angiogenesis entails the development of new blood vessels from established vascular beds and as such is different from vasculogenesis (de novo formation of vessels from bone marrow-derived endothelial precursor cells) or vasculogenic mimicry (the ability of tumor (stem) cells to form vessel-like networks). An angiogenic state should be reverted back to quiescence after the physiological challenge has been overcome. Instead, pathological angiogenesis in tumors is fueled by a continuous imbalance between pro- and anti-angiogenic signaling in the TME, mainly driven by the VEGF-VEGFR2 axis [79, 80] (Fig. 3). Thus, tumor-residing ECs are subjected to an abundance of VEGF, nutrient-deprivation (i.e. hypoxia and low glucose) and aberrant blood flow. These harsh TME conditions drive uncontrolled vessel sprouting and affect the vessel function and maturation. In addition, tumor vessels are associated with high permeability and low maturation/stability. These structural and functional abnormalities of the vessel wall result in tumor-associated vessels that are structurally weak with varying diameters (prone to collapse due to e.g. mechanical pressure from proliferating tumor cells) and do not comply with a structured hierarchy, which lead to blood flow disruptions and inadequate perfusion of oxygen/nutrient-rich blood. These vascular abnormalities ultimately foster hypoxia, nutrient deprivation, acidity, and inflammation, which are all TME-related factors promoting tumor growth and cancer cell dissemination. ECs embedded in the TME are thus exposed to stressful conditions that typically result in heightened autophagic flux. Indeed, tumor-associated ECs upregulate autophagy compared to normal ECs that at least mediates resistance to hypoxia-induced cell death [81].

Yet, if autophagy or the endo-lysosomal system maintain or worsen EC function in tumors, modify the interface with cancer cells and other stromal cells and/or influence tumor dissemination and therapeutic outcome, is currently a looming field.

### Autophagy and endo-lysosomal system in tumor angiogenesis

The compromised vascular barrier integrity observed in tumor vessels is a consequence of disrupted adherence and tight junctions between neighboring ECs commonly due to hyper-activation of VEGF signaling. Indeed, in the endothelium, increased permeability is associated with a decreased expression of cell surface CDH5. Of note, blockade of lysosomal degradation by CQ in vivo augments CDH5 endothelial junctions and pericyte coverage of tumor vessels, resulting in tightening of the EC barrier in vivo [62]. This is likely the reason why cancer cell intravasation and the number of circulating cancer cells is decreased by CQ in this tumor model. Notably, although genetic loss of *Atg5* in ECs delays melanoma growth, it does not affect metastasis and tumor oxygenation. On the contrary, genetic depletion of *Atg5* in ECs worsens both structural and functional features of the vessels, exacerbating the chaotic and functionally abnormal tumor vasculature [62]. This underscores that the effects of pharmacological blockade of lysosomal function or genetic inhibition of key autophagy mediators, such as ATG5, have opposite effects on the tumor vasculature, which might have important consequences on therapeutic application, as discussed further in the next sections.

However, beyond its beneficial role in keeping redox homeostasis and EC permeability in check, heightened autophagy in TECs could also support the increased metabolic needs of the hyper-proliferating TECs and enhance adaptation to the metabolic stressors of the TME. For example, hypoxia is a known inducer of autophagy and tumor-associated ECs are more resistant to hypoxia-induced cell death than normal ECs [81]. Hypoxia in ECs not only drives autophagy but also stabilizes the α-subunit of the hypoxia-induced factor (HIF)-complex, which will stimulate the expression of VEGF and glycolytic genes. Of note, tumors from *Becn1*^*+*^^*/−*^ mice show heightened angiogenic potential compared to their WT counterparts under hypoxia. Moreover, ECs isolated from *Becn1*^+/−^ mice show increased proliferation, migration, and tube formation potential in response to hypoxia relative to WT cells. The enhanced EC tip-like phenotype caused by heterozygous loss of *Becn1* is dependent on the elevation in HIF-2α expression relative to HIF-1α in response to hypoxia. This is reflected by an increased production of erythropoietin a target of HIF-2α [82] and possibly other pro-inflammatory factors. Altogether these studies support the view that autophagy in tumoral ECs may confine—albeit not suppress—excessive angiogenesis. However, given that ATG5 and BECN1 regulate other pathways beyond canonical autophagy [83], it would be important to further evaluate the effects of the EC-specific genetic deletion of other autophagy mediators on tumor vessels and tumor growth, to get further insights into the role of autophagy in this crucial stromal compartment.

As mentioned above, angiogenesis imposes to ECs a metabolic shift to meet the augmented energy demand for migration and proliferation. However, whether autophagy fuels the high energy demand of these hyper-proliferating TECs, remains elusive. This hypothesis moreover contrasts with the pro-angiogenic phenotypes observed in vivo upon EC-specific deletion of *Atg5*, or in *Becn1*^+/-^ hemizygous mice described above. It is therefore possible that autophagy favors metabolic rewiring toward an EC quiescent phenotype, by maintaining mitochondria health (via mitophagy) and degrading lipid droplets. These processes combined, would support FAO while keeping ROS levels in check.

Besides these cell-autonomous processes, autophagy and more in general the endo-lysosomal system in TECs could also be involved in the regulation of the expression of cell surface factors and secretion of cytokines/chemokines involved in the interface with cancer and other stromal cells. As discussed previously, autophagy has emerging functions in (unconventional) secretion of cytokines [14] and angiogenesis-related factors [64, 84]. Moreover, within the inflamed TME, ECs may change their secretome and how autophagy/lysosomal pathway may modulate this process is still understudied. For example, in ECs autophagy contributes to the secretion of high mobility group box 1 (HMGB1), which is highly upregulated in the tumor endothelium [85]. Aside from its inflammatory role, HMGB1 also functions in tissue remodeling and angiogenesis [86]. Interestingly, HMGB1 is involved in resistance toward tumor vessel-targeted, monoclonal antibody-based immunotherapy [87]. The release of type I interferon (IFN) predominantly by tumoral ECs favors cytotoxic T-cell-mediated antitumor immunity in a melanoma mouse model [88], thus further indicating that the tumor endothelium is not just a passive player, but actively contributes in modifying the cross talk between stromal cells and cancer cells. Interestingly, in fibroblasts autophagosome trafficking modulates IFN-β production [89], thus raising the interesting possibility that this process may be regulated by autophagy TEC as well.

In conclusion, based on the limited studies available addressing the effects of genetic ablation of autophagy genes in ECs on the TME and tumor growth, it is tempting to propose that autophagy in TECs may support both cell-autonomous (degradation, metabolism, and redox control) and non-autonomous (trafficking and secretion) functions of the aberrant and inflamed endothelium.

### Vessel normalization and the endo-lysosomal system

The abnormal tumor vasculature results in poor tumor perfusion, which not only favors cancer genetic instability, dissemination, and metastasis but also compromises drug delivery. Additionally, hypoperfused/hypoxic tumors have impaired T-lymphocyte function and worse prognosis compared to their normoxic counterparts [90]. In accordance, conditions and strategies alleviating tumor hypoxia represent an important aim in anticancer therapy [91, 92].

**Fig. 4:**
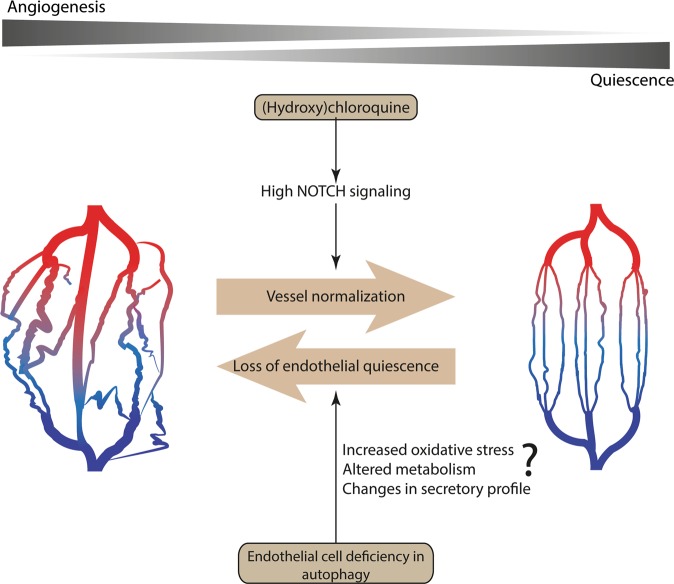
Genetic blockade of autophagy in endothelial cells (ECs) fosters tumor angiogenesis while systemic treatment with chloroquine (CQ) induces vessel normalization. Left: tumor vasculature is in a state of continuous remodeling due to imbalanced pro- and anti-angiogenic signaling in the tumor microenvironment. CQ treatment induces (right) vessel normalization in tumors, mainly by activating NOTCH1 signaling pathway during its endocytic route. Enhanced NOTCH1 signaling promotes the quiescent EC phenotype. As a result, vessel functionality and structure are improved. Autophagy is heightened in tumor-associated ECs as an adaptive response to overcome metabolic stress and/or as an attempt to reinstate quiescence through reducing oxidative stress. EC-specific genetic impairment of autophagy is associated with a highly angiogenic vascular phenotype. These differential effects on EC function should be taken into consideration when devising interventions aiming to modulate autophagy or the endo-lysososmal system in anticancer therapy

The initial strategy for vessel-targeting therapy, aimed to inhibit new vessel formation and to destroy the tumor vasculature, thereby starving the tumor cells by reducing nutrient provision. However, anti-angiogenic therapies have been less successful than initially hoped for. This is largely caused by the induction of hypoxia (leading to resistance to chemotherapy or recruitment of myeloid cells that bypass tumor-inhibitory effects of therapy [93]) and increased vascular permeability (thereby increasing tumor cell intravasation) [94–97]. In line with this, targeting tumor-associated ECs using monoclonal CD31 antibodies showed that the resulting hypoxia induces several changes in tumor cells, including increased epithelial-mesenchymal transition and vascular mimicry-related gene expression, allowing them to escape the anti-angiogenic therapy [98]. Accumulating evidence instead favor strategies causing a normalization of the tumor vasculature (Fig. 4). By normalizing the tumor vasculature, instead of pruning it, vessel functionality and perfusion are ameliorated, and as a consequence tumor hypoxia is reduced while transporting capability of vessels are improved, resulting in a better drug delivery and therapeutic responses [95].

Of note, inhibition of PFKFB3 induces vessel normalization [99, 100] suggesting that inhibition of glycolysis in TECs is sufficient per se to normalize the tumor vasculature, at least temporally. Interestingly, also inhibition of lysosomal function through CQ treatment of melanoma-bearing mice induces vessel normalization (Fig. 4) [62]. The in vivo effects of CQ on tumor oxygenation, vessel normalization, and metastasis are reverted by the specific deletion of NOTCH1 in ECs. Since NOTCH1 activation leads to repression of *PFKFB3* transcription [49] this signaling axis could contribute to the vessel normalizing effect of CQ [62].

A two-way cross talk exists between immune cells and vasculature that impacts vessel maturation/function. Notably, myeloid-derived suppressor cells (MDSCs) and tumor-associated macrophages render tumors ill-responsive to VEGF/VEGFR inhibition [93] and to (active) CD4^+^ T cells that promote vessel normalization [101]. A recent study shows that CQ facilitates antitumor T-cell immunity by repolarizing tumor-promoting M2 macrophages in the TME to tumor-inhibiting M1 macrophages [102], which can be an additional mechanism explaining the vessel normalization effects of this lysosomotropic drug [103]. In macrophages, CQ-induced effects are dependent on lysosomal calcium release [102]. In addition, the CQ-mediated reduction in tumor hypoxia [62] may favor a less pro-angiogenic TME as hypoxia stimulates the M2 phenotype and MDSC expansion [104, 105]. CQ has demonstrated mild immunosuppressive effects supported by its clinical application in the treatment of certain autoimmune diseases such as rheumatoid arthritis. Yet, it is possible that its normalizing effects on ECs (and thereby vascular structure/function), leading to a less hypoxic TME, might overcome its reported negative influence on T cells thus indirectly favoring antitumor immunity [106]. Preclinical studies show that treatment with CQ or its derivative hydroxychloroquine (HCQ) has no major antagonist effect on antitumor-mediated T-cell responses, suggesting that these lysosomotropic drugs may be used not only in combination with chemotherapies but also with immunotherapies [107]. Also whether different CQ derivatives with enhanced bioactivity and improved antitumoral efficacy [108–110] will retain the tumor vessel-normalizing effects of CQ and/or have other effects on stromal cells and T-cell-mediated antitumor immunity still needs to be addressed. The search for chemical modulators or inhibitors of specific autophagy-related proteins should be pursued as well. As such it should be noted that results obtained by genetic interference (e.g. knockdown or knockout) of *Atgs* do not necessarily and always comply with pharmacological inhibition of the endo-lysosomal system (e.g. by CQ/HCQ treatment). For example, sensitivity to radiotherapy of cancer cells is affected by ATG7 and LC3B knockdown, but not by CQ treatment [111] and as discussed above, CQ, but not EC-specific ATG5 deficiency, leads to vessel normalization by activating the NOTCH1 signaling pathway during its endocytic route [62]. Moreover, just like for CQ/HCQ, more specific chemical inhibitors of autophagy proteins, like ATG4 [112], should be tested for their in vivo anticancer activity considering their potential impact not only on cancer cells, but on stromal cells as well.

In conclusion, interference with the endo-lysosomal pathway by CQ is an efficacious strategy to reduce pathological angiogenesis and induce vessel normalization in tumor. Delineating why depletion of ATG5 or other key autophagy proteins in TECs instead further aggravates tumor angiogenesis is mandatory to define a role for autophagy in this crucial stromal compartment (Fig. 4).

## Perspectives

Our understanding of the role and functional consequences of EC-associated autophagy in physiological and pathological angiogenesis is still in its infancy. In spite of this, it is becoming clear that EC-intrinsic autophagy influences several aspects of the EC biology and vasculature functions (e.g. survival in response to metabolic stressors, redox homeostasis, vessel permeability, and secretion of blood clotting factors). On the other hand, it remains elusive whether and how in the tumor, EC autophagy modulates EC properties thereby crucially influencing angiogenesis and cancer growth. An even more blurry area is how autophagy in ECs interfaces with other stromal components of the tumor, such as immune cells, thereby modulating key processes like immunosurveillance and responses to anticancer immunotherapy. From a cell-autonomous view, autophagy could dynamically support the plasticity of ECs by regulating redox homeostasis and metabolic rewiring, whose relevance for EC biology, the aging process, and tumor angiogenesis is becoming increasingly recognized. Moreover, autophagy has emerging non-cell-autonomous roles, e.g. in secretion, thereby controlling autocrine/paracrine signals. Given this complexity, to further untie the role of EC-associated autophagy in cancer, more in vivo and cellular studies targeting a different subset of autophagy regulators are needed.

From a therapeutic perspective, since it is emerging that both autophagy and endo-lysosomal system have major and often not overlapping impact on EC function and that the maturation state of vessels influences outcome of adjuvant treatment, it is important to further reveal how these mechanisms operate and interface with other key EC-related processes. In most preclinical studies, pharmacological inhibition of autophagy is often based on drugs that impair lysosomal function such as the clinically used, Food and Drug Administration-approved CQ/HCQ, either alone or in combination with chemotherapy, kinase inhibitors, or radiotherapy [113]. Clinical studies have shown that CQ/HCQ have a good safety profile, and in most cases increase, albeit still marginally, the clinical efficacy of the combined treatments [67]. However, we still have a poor understanding on the effects of these drugs on the tumor stroma in clinical settings, due largely to the absence of proper biomarkers, and of their therapeutic window in order to devise the most efficient treatment schedule. Finally, there is an urgent need to improve current therapies targeting pathological angiogenesis based on the VEGF signaling blockade, since these interventions have limited success and are prone to refractoriness and resistance [93, 114]. Interventions eliciting vessel normalization, improve tumor perfusion, drug delivery, and therapy responses are thought to offer an alternative and more efficacious option [49, 115].

Altogether these insights will uncover the effects of autophagy-endo-lysosomal modulating therapies on ECs alongside other tumor entities (e.g. cancer cells and immune cells) to further develop a combinatory therapy that is effective in tumor control.
